# Prolactinomas: Prognostic Factors of Early Remission After Transsphenoidal Surgery

**DOI:** 10.3389/fendo.2020.00439

**Published:** 2020-07-07

**Authors:** Grzegorz Zielinski, Marcin Ozdarski, Maria Maksymowicz, Katarzyna Szamotulska, Przemysław Witek

**Affiliations:** ^1^Department of Neurosurgery, Military Institute of Medicine, Warsaw, Poland; ^2^Military Outpatient Clinic, Nowy Dwór Mazowiecki, Poland; ^3^Department of Pathology and Laboratory Diagnostics, Maria Curie-Skłodowska Memorial, National Institute of Oncology, Warsaw, Poland; ^4^Department of Epidemiology and Biostatistics, Institute of Mother and Child, Warsaw, Poland; ^5^Department of Gastroenterology, Endocrinology and Internal Diseases, Military Institute of Medicine, Warsaw, Poland; ^6^Department of Internal Diseases, Endocrinology and Diabetes, Medical University of Warsaw, Warsaw, Poland

**Keywords:** prolactinoma, dopamine agonist, transsphenoidal surgery, pituitary adenoma, PRL, plurihormonality

## Abstract

**Background and Objective:** Most patients with prolactinomas receive pharmacological treatment only, resulting in limited research on the predictors of successful prolactinoma surgery. In this study, we analyzed whether early postoperative serum prolactin concentrations and selected tumor characteristics could predict early, hormonal remission after removal of prolactinomas.

**Methods:** We prospectively enrolled 48 consecutive patients with prolactinomas who underwent transsphenoidal resection performed by the same surgeon. Early remission, defined as a lack of hyperprolactinemia symptoms and normalization of serum prolactin concentration, was ascertained in all patients at 3 months. We evaluated the invasiveness of prolactinomas on the Knosp grading scale and measured serum prolactin concentrations on the first postoperative day. Routine immunohistochemical analysis, evaluation for plurihormonality, and assessment of the Ki-67 proliferation index (<3 or ≥3% of positive nuclei) were performed in all tumor samples.

**Results:** Of 48 patients, 38 (79%) achieved early biochemical remission at 3 months. Patients in early remission at 3 months had lower serum prolactin concentrations on the first postoperative day than patients with recurrent or persistent hyperprolactinemia (*p* < 0.001). Using univariate logistic regression, larger maximum tumor diameter (*p* = 0.014), higher Knosp grade (*p* < 0.001), and plurihormonality predicted remission at 3 months (*p* = 0.021). However, using multivariate stepwise logistic regression, only the Knosp grade remained significant (*p* < 0.001).

**Conclusions:** Radiological assessment of prolactinoma invasiveness (Knosp grades) and early postoperative serum prolactin concentrations are important predictors of early remission following transsphenoidal prolactinoma resection.

## Introduction

Prolactinomas are the most common pituitary tumors, accounting for about 60% of hormone-secreting pituitary tumors (1, 2). Typically, patients with prolactinomas present symptoms of hyperprolactinemia: women present with menstrual dysfunction and galactorrhea and men present with sexual dysfunction and gynecomastia. In men and postmenopausal women, symptoms of hyperprolactinemia are recognized late; these patients tend to have larger or more invasive prolactinomas, which may cause headaches, loss of vision, or cranial nerve palsies (3, 4).

Dopamine agonists are the first-line treatment for prolactinomas because of their effectiveness in about 80% of patients (5–8). Dopamine agonists alleviate the symptoms of hyperprolactinemia, slow tumor growth, and sometimes even decrease tumor size. However, when dopamine agonists are ineffective or cause inacceptable adverse effects, endoscopic transsphenoidal prolactinoma resection is recommended. This type of surgery is safe and effective, with remission rates of 50–90% (2, 9–13).

Previous studies suggested that men, elderly patients, patients with large, invasive, or highly proliferating tumors, and patients with high post-operative prolactin concentrations or previous incomplete resections are less likely to achieve remission after surgical removal of prolactinomas (14–19). However, predicting outcomes of prolactinoma resection based on the available evidence remains difficult (20). Therefore, we analyzed which of the selected clinical, imaging, pathological, and hormonal characteristics were associated with early remission after transsphenoidal removal of prolactinomas in our patients.

## Materials and Methods

### Patients

We prospectively enrolled 48 consecutive patients with prolactinomas who underwent transsphenoidal resection between 2013 and 2016 either because of resistance to or poor tolerance of medical therapy. Patients were operated on by the same neurosurgeon according to the same surgical protocol. We recorded baseline demographic and clinical characteristics of patients and surgery complications. We assessed remission rates at 3 months and long-term, i.e., over a mean follow-up of 82.2 ± 34.6 months [± standard deviation (SD)].

### Tumor Invasiveness

All patients underwent magnetic resonance imaging (MRI) of the pituitary (GE Signa, 1.5 Tesla) before and after intravenous injection of gadolinium (Gd-DTPA). Tumor size was assessed by measuring craniocaudal, transverse, and anteroposterior dimensions. The largest of the three was considered representative of the tumor size (RECIST criteria). Prolactinomas were classified as microprolactinomas (diameter ≤ 10 mm) or macroprolactinomas (diameter > 10 mm). Cavernous sinus invasiveness was determined by image analysis of coronal MRI according to the Knosp criteria (21). Tumors with no evidence of invasion were considered to be Knosp grade 0 or 1, while tumors with evidence of invasion were graded as Knosp 2–4.

### Surgical Procedure

All procedures were performed under general anesthesia with endotracheal intubation using binostril, three-hand technique. A 0°, or less frequently 30° (in cases of adenomas invading cavernous sinus), 30 cm in length rigid endoscopes with a lens diameter of 4 mm and an automatic endoscope irrigating system (Karl Storz, Tuttlingen, Germany) were used. A wide anterior sphenoidotomy and partial posterior septectomy without middle turbinectomy was performed with Kerrison rongeur and/or high-speed drill to get a wide exposure of the sellar floor. Afterwards, the sellar floor was opened with a high-speed drill. The dural opening with microscissors was made and the selective adenomectomy was performed using microsurgical technique (microdissection, curettage, and suction). The preferred technique of the tumor removal was gentle dissecting adenoma pseudocapsule from the pituitary gland and the medial wall of the cavernous sinus (for laterally placed lesions). Detailed endoscopic exploration of the tumor bed (with straight and angled lens endoscope) with special reference for remnants of the adenoma in close proximity of the medial wall of the cavernous sinus was performed at the end of each operation. Fibrin glue was applied for the reconstruction of the sellar floor. Neither fluoroscopy nor frameless stereotactic image guidance or intraoperative MR imaging were utilized.

### Hormonal Assessments and Remission Criteria

Serum prolactin concentrations were measured with an electrochemiluminescence immunoassay (Elecsys, Roche Diagnostics). The limit of detection was 0.047 ng/mL. The normal range was 4.04–15.2 ng/mL for men and 4.79–23.3 ng/mL for women. Remission was defined as a lack of hyperprolactinemia symptoms and normalization of serum prolactin concentration.

### Pathological Examination

Surgical specimens from all patients were analyzed according to the WHO 2004 guidelines using hematoxylin and eosin staining as well as immunohistochemistry (22)—this classification was valid for the period from which the presented cases originate. Immunohistochemical staining was performed on paraffin-embedded specimens according to the labeled EnVision Flex Visualization System (Dako, K8000) with DAB as chromogen using antibodies against anterior pituitary hormones: prolactin (PRL, 1:200), growth hormone (GH, 1:500), adrenocorticotrophic hormone (ACTH, 1:500), thyroid-stimulating hormone (β-TSH, 1:500), follicle-stimulating hormone (β-FSH, 1:500), luteinizing hormone (β-LH, 1:500), all antibodies from Thermo Scientific Lab Vision Corp., and glycoprotein α-subunit (1:100) from Novocastra. Ki-67 (MIB-1) from Dako (ready to use) were used. Histopathological grading was based on the revised WHO classification system of tumors of endocrine organs (22).

For the purpose of our study, tumors stained for prolactin and the alpha subunit or pituitary hormones other than prolactin, or both, were classified as plurihormonal. Tumors that stained for prolactin only were classified as pure lactotroph tumors. We assessed the proliferative activity by calculating the percentage of tumor cells with nuclear Ki-67 expression. The Ki-67 (MIB-1) labeling index was graded in two categories: <3 or ≥3% of positive nuclei. All cases were also assessed in the Philips BioTwin CM 120 Transmission Electron Microscope.

### Statistical Analysis

We used descriptive statistics in accordance with the distribution of variables. The chi-squared, Fisher's exact, McNemar, *t*-Student, and Mann–Whitney tests were used for comparisons. The Pearson correlation coefficient was used to study associations between continuous variables. Logarithmic transformation of variables was applied where appropriate. Univariate logistic regression and multivariate stepwise forward logistic regression were used to find significant predictors of remission. *P* < 0.05 was regarded statistically significant. All calculations were completed in the IBM SPSS v. 25 (IBM, USA).

## Results

Baseline characteristics of all 48 patients, including age, gender, hormonal assessment, previous medical treatment, and results of pathological examinations are presented in Table 1. Sparsely granulated lactotroph adenoma was diagnosed in all patients. Of 48 patients, 38 (79%) achieved biochemical remission at 3 months.

**Table 1:** Baseline characteristics.

**Variable**	**Value**
Age, years (mean ± SD)	30.1 ± 9.2
Women, *n* (%)	44 (92)
**Symptoms**, ***n*** **(%)**	
Primary amenorrhea (females)	5(11)
Secondary amenorrhea (females)	39 (89)
Vision disturbances	3 (6)
Headache	18 (38)
Decreased libido (males)	4 (100)
Symptom duration, years [median(IQR)]	5.0 (3.0–7.0)
**Medical treatment**, ***n*** **(%)**	
Bromocriptine	36 (75)
Quinagolide	14 (29)
Cabergoline	34 (71)
≥2 medications	28 (58)
Median (IQR) serum prolactin concentration before surgery, μg/dL	232.5 (151.8–407.5)
Microprolactinomas (≤ 10 mm), *n* (%)	26 (54)
Maximum tumor diameter, mm (median(IQR))	10.0 (9.0–15.0)
**Knosp grade**, ***n*** **(%)**	
0	19 (40)
1	19 (40)
2	4 (8)
3	3 (6)
4	3 (6)
**Ki-67 expression**, ***n*** **(%)**	
<3%	32 (74)
≥3%	11 (26)
Tumor hormone secretion, *n* (%)	
Pure lactotroph	35 (81)
Plurihormonal	8 (19)^a^
Ultrastructure: Sparsely granulated tumors (SG-PRL), *n* (%)	48 (100)

*IQR, interquartile range; SD, standard deviation; ^a^alpha-SU(+) (n = 5), ACTH(+) (n = 1), GH(+) (n = 1), LH(+) (n = 1)*.

**Figure 1:**
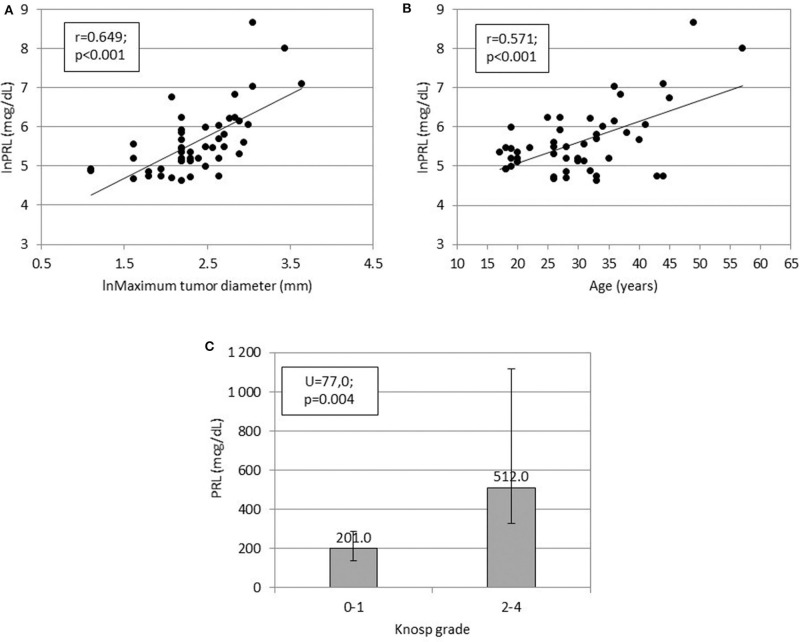
Relationships between **(A)** serum prolactin concentration (ln) and prolactinoma size (ln), **(B)** serum prolactin concentration (ln) and patients' age, and **(C)** serum prolactin concentration and Knosp grade.

Preoperative serum prolactin concentrations were positively associated with maximum tumor diameter (*r* = 0.649, *p* < 0.001), Knosp grade (*U* = 77.0, *p* = 0.004), and patient age (*r* = 0.571, *p* < 0.001, Figure 1). Compared to microadenomas, macroadenomas had higher Knosp grades (*p* = 0.006, Table 2) and were more often plurihormonal (7/22, 32% vs. 1/21, 5%; *p* = 0.046). Plurihormonal and pure lactotroph tumors did not differ significantly with respect to invasiveness: the proportion of Knosp grade 2–4 tumors was 5/35 (14%) for pure lactotroph tumors and 3/8 (38%) for plurihormonal tumors (*p* = 0.153). Similarly, we did not confirm the relationship with gender: 7 of 8 plurihormonal tumors (88%) and 32 of 35 pure lactotroph tumors (91%) were found in women; (*p* = 1.000). In addition, plurihormonality was not related to age: of 7 plurihormonal tumors, 1 occurred in patients aged ≤ 24 years (14%), 3 in patients aged 25–34 (43%), and 3 in patients aged ≥ 35 (43%). Ten of 32 pure lactotroph tumors occurred in patients aged ≤ 24 years (31%), 16 in patients aged 25–34 (50%), and 6 in patients aged ≥ 35 (19%, *p* = 0.355). Symptom duration before surgery was associated with age (*r* = 0.495, *p* < 0.001), but it was not related to preoperative prolactin concentrations (*r* = 0.275, *p* = 0.059) or to maximum tumor diameter (*r* = 0.212, *p* = 0.149). Maximum tumor diameter correlated with age (*r* = 0.316, *p* = 0.029).

**Table 2:** Knosp grade of macroprolactinomas and microprolactinomas.

	**Knosp grade**				
	**0**	**1**	**2**	**3**	**4**
Microprolactinomas, *n* (%)	16 (62)	7 (27)	1 (4)	1 (4)	1 (4)
Macroprolactinomas, *n* (%)	3 (14)	12 (54)	3 (14)	2 (9)	2 (9)
All prolactinomas, *n* (%)	19 (40)	19 (40)	4 (8)	3 (6)	3 (6)

*p = 0.006 (Fisher's exact test)*.

We confirmed the relationship between higher proliferation index (Ki-67) and preoperative serum prolactin concentration (median: 270 vs. 191 ng/mL; *p* = 0.048). The borderline association was also found between higher category of Ki-67 and higher Knosp grade or plurihormonality (0.079 and 0.079, respectively).

**Figure 2:**
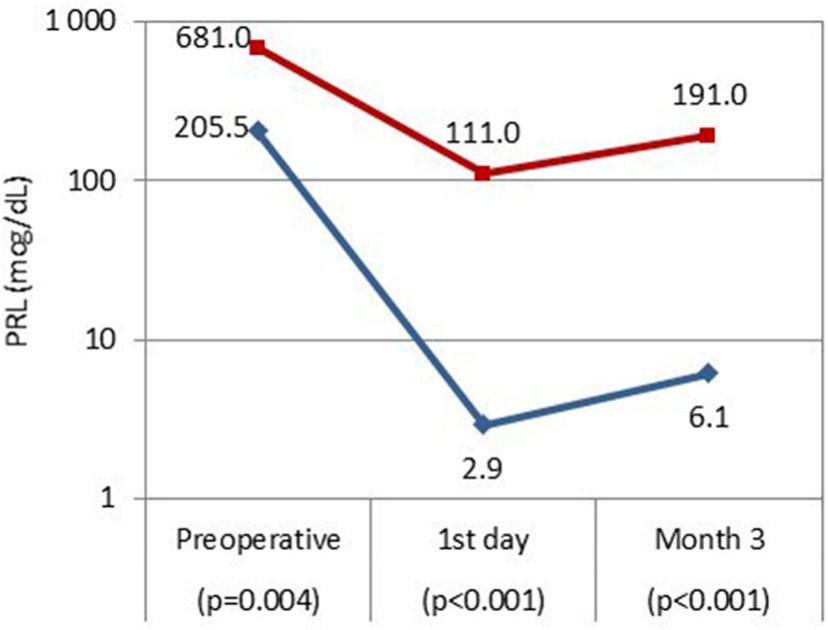
Serum prolactin concentrations before transsphenoidal surgery, on the 1st postoperative day and 3 months after surgery in patients with remission (blue line) and without remission (red line).

Among all patients, the median (interquartile range) prolactin concentration on the 1st postoperative day was 3.4 (1.23–13.73) ng/mL, and was 10.66 (4.12–18.75) ng/mL 3 months after surgery. Patients in early remission had significantly lower serum prolactin concentrations on the 1st postoperative day (*p* < 0.001) and at 3 months after surgery (*p* < 0.001) as compared to patients without remission (Figure 2). The proportions of prolactin concentration within referral range did not differ between 1st postoperative day and 3 months after surgery: nearly all patients with normal serum prolactin concentrations 1 day after surgery maintained remission at 3 months (37/38, 97%), and among patients with increased prolactin concentration on the 1st postoperative day only 1 achieved remission at 3 months (1/10, 10%; *p* = 1.000).

Patients who achieved remission at 3 months had smaller adenomas (median: 9.5 mm) than the remaining patients (median: 15.5 mm, *p* = 0.032).

The remission rate at 3 months was higher in patients with Knosp grades 0–1 (36/38, 95%) than in those with Knosp grades 2–4 (2/10, 20%; *p* < 0.001). This was similar to patients with Ki-67 <3% (27/32, 84%) and ≥ 3% (8/11, 73%, *p* = 0.401), and higher in patients with pure lactotroph tumors (31/35, 89%) than in those with plurihormonal tumors (4/8, 50%; *p* = 0.028). Importantly, none of the patients with Knosp grades 3–4 (*n* = 6) achieved remission at 3 months.

On univariate logistic regression, maximum tumor diameter (*p* = 0.014), Knosp grade (0–1 vs. 2–4, *p* < 0.001), and plurihormonality (*p* = 0.021) predicted remission at 3 months but on multivariate stepwise logistic regression, only the Knosp grade remained significant (*p* < 0.001; Table 3). The adjustment for baseline values of serum PRL concentration did not influence the results of logistic regression.

**Table 3:** Predictors of remission 3 months after prolactinoma resection.

**Predictor**	**Univariate logistic**		**Multivariate stepwise**	
	**regression**		**logistic regression**	
	**OR (95% CI)**	***P***	**OR (95% CI)**	***p***
Knosp 2–4 vs. Knosp 0–1	72.0 (8.8–590.5)	<0.001	49.500 (5.8-422.5)	<0.001
Maximum tumor diameter (mm)	1.2 (1.0–1.4)	0.014	–	–
Plurihormonal vs. pure lactotroph tumors	7.8 (1.4–43.9)	0.021	–	–

*CI, confidence interval; OR, odds ratio*.

At the end of a mean follow-up of nearly 7 years, 34 patients maintained remission (71%); none of the patients who were not in remission at 3 months achieved long-term remission.

We confirmed one case of secondary adrenal insufficiency (2%) and two cases of secondary hypothyroidism (4%) which required hormonal replacement therapy. No patient developed postoperative hyponatremia due to inappropriate antidiuretic hormone secretion (SIADH), or persistent diabetes insipidus. There was no postoperative cerebrospinal fluid leakage or meningitis in our group.

## Discussion

We found that parasellar invasion was a significant predictor of both short- and long-term remission after endoscopic transsphenoidal prolactinoma resection, and prolactinoma size was not related to surgery outcomes independently of parasellar invasion. Plurihormonal prolactinomas and prolactinomas with an increased proliferative activity (Ki-67 index ≥ 3%) were not associated with worse long-term outcomes. In our group of patients with prolactinomas, transsphenoidal resection was safe and effective with no deaths or cases of diabetes insipidus related to surgery.

The patients with prolactinomas included in our analysis required surgery because of unsatisfactory effects of pharmacological treatments. Such patients are rare, as most patients with prolactinomas receive pharmacological treatment successfully. Thus, few studies have investigated prognostic factors of remission after surgical removal of prolactinomas, and one-center reports in which all patients were operated on by the same surgeon are even rarer. Because of scarce evidence, it has been unclear whether markers of proliferation are useful in assessing the aggressiveness of prolactinomas. In the 2017 WHO classification, Ki-67 indices ≥ 3% are considered to indicate aggressive pituitary tumors (23). It should be underlined that the pathological results of our patients are not representative for patients with prolactinoma at large, but rather for patients in whom drug treatment failed. Drug resistance was reported to be one of the predictors of poor prognosis in patients with prolactinoma (24).

Despite this fact, more than 70% of our patients achieved remission after surgical resection, which is in line with published work (9–11, 25). Similar to previous studies, we found that the remission rate was higher in patients with microprolactinomas than in those with macroprolactinomas (25–28). Moreover, larger tumor size was associated with a lower likelihood of remission after surgery. However, tumor size was not significantly associated with remission when corrected for parasellar invasion. Similar to our findings, Raverot et al. (18) reported that prolactinoma invasiveness, but not size, was a predictor of outcomes after prolactinoma surgery. However, other investigators found both prolactinoma size and invasiveness as independent predictors of the outcomes of prolactinoma surgery (19). Our findings suggest that prolactinoma invasiveness assessed radiologically is a good indicator of tumor aggressiveness. Indeed, none of the patients with Knosp grades 3–4, which indicates cavernous sinus invasion (21), achieved remission after surgery.

In our study, patients with early biochemical remission had significantly lower preoperative serum prolactin concentrations as compared to patients without remission. In previous works, preoperative prolactin concentrations were not associated with long-term outcomes (16, 18). In contrast, serum prolactin concentrations measured immediately after prolactinoma removal were important for predicting remission: those in remission had significantly lower prolactin concentrations on the 1st postoperative day. Similarly, in another study, higher postoperative prolactin concentrations were associated with a greater risk of surgery failure (16). Moreover, Amar et al. (27) showed that nearly all patients with prolactinomas and prolactin concentrations <10 ng/mL measured 1 day after surgery achieved remission (100% of patients with microprolactinomas, 93% of patients with macroprolactinomas). Such findings are likely resections, which are associated with worse outcomes. Similarly, low, early postoperative concentrations of cortisol predict the success of tumor removal in patients with Cushing's disease (29, 30). It remains unclear, however, what cut-off values of prolactin concentrations to adopt. In case of CD and serum cortisol usually so called “sub-normal values” (i.e., <2.0 μg/dL) are adopted (29, 30). In this study, we assumed the upper limit of referral range. Larger studies are needed to establish reliable cut-off concentrations that can be used in clinical practice.

Similar to other investigators, we showed that preoperative serum prolactin concentrations correlated positively with prolactinoma size (15, 31), tumor invasiveness (15, 32), and patient age (33). The positive relationship between age and prolactin concentrations may be explained by larger tumors observed in older patients than in younger individuals (33).

Plurihormonal adenomas are considered as more invasive (higher Ki-67 indices) and more prone to early recurrences than monohormonal adenomas (34–37). In our study, plurihormonal prolactinomas were larger but not more invasive than lactotroph tumors. Moreover, plurihormonality was not identified as an independent risk factor for surgical failure, probably because it was not related to tumor invasiveness. Plurihormonality may be important for predicting surgery outcomes in patients with large prolactinomas (>4 cm), however, we did not have patients with such large tumors in our study. We believe that tumor invasiveness is a better prognostic factor than other tumor characteristics. When the tumor has low invasiveness, it can be removed completely, which makes other tumor characteristics less important.

In the previous studies, Ki-67 values > 3% were associated with greater microscopic prolactinoma invasiveness (38, 39). These studies also showed a higher Ki-67 labeling index (LI) in atypical lactotroph adenomas (mean value of 7.2%) and, remarkably, this adenoma subtype presented the greatest local invasiveness (40). In our study, patients with or without remission had tumors with similar proliferative activity (Ki-67 indices), similar to data published by Grimm et al. (41).

In another study, invasive and noninvasive prolactinomas, as assessed radiologically, also had similar Ki-67 indices (15). Thus, radiological and microscopic markers of prolactinoma invasiveness seem unrelated.

In statistical terms, our sample was relatively small, however, because most patients with prolactinomas receive drug treatment only, this limitation occurs often in studies that assess outcomes of prolactinoma surgery. The follow-up was relatively short, while sometimes prolactinomas recur a dozen years after surgery. The strength of our study was the prospective inclusion of a homogenous group of patients, all from one center and operated on by the same surgeon, who received a rare treatment option for prolactinomas. In our study, prolactinoma samples were examined with standard histopathological methods and future studies could benefit from additional histological and molecular data (18).

In conclusion, radiological assessment of prolactinoma invasiveness (Knosp grades) and early postoperative serum prolactin concentrations are important for predicting outcomes of transsphenoidal prolactinoma resection. Transsphenoidal resection of prolactinomas is safe and effective in patients with non-invasive adenomas who do not respond to or tolerate dopamine agonists.

## Data Availability Statement

The raw data supporting the conclusions of this article will be made available by the authors, without undue reservation.

## Ethics Statement

The studies involving human participants were reviewed and approved by Bioethics Committee of the Military Institute of Medicine. The patients/participants provided their written informed consent to participate in this study.

## Author Contributions

GZ, PW, MO, and MM designed the study. PW, GZ, MM, and MO collected the data. PW, GZ, MO, MM, and KS contributed to data analysis and interpretation. PW and GZ drafted the manuscript. All authors contributed to the revision and approval of the final version of the paper.

## Conflict of Interest

The authors declare that the research was conducted in the absence of any commercial or financial relationships that could be construed as a potential conflict of interest.
